# A blaschkoid birthmark

**DOI:** 10.1016/j.jdcr.2024.02.002

**Published:** 2024-02-13

**Authors:** Miranda Tomaras, Margaret S. Newsome, Morgan Thakore

**Affiliations:** a School of Medicine, Baylor College of Medicine, Houston, Texas; b Department of Dermatology, Wellstar MCG Health, Augusta, Georgia

**Keywords:** birthmark, blaschkoid, congenital, nevi, nevus comedonicus, pediatric

## History

**Fig 1:**
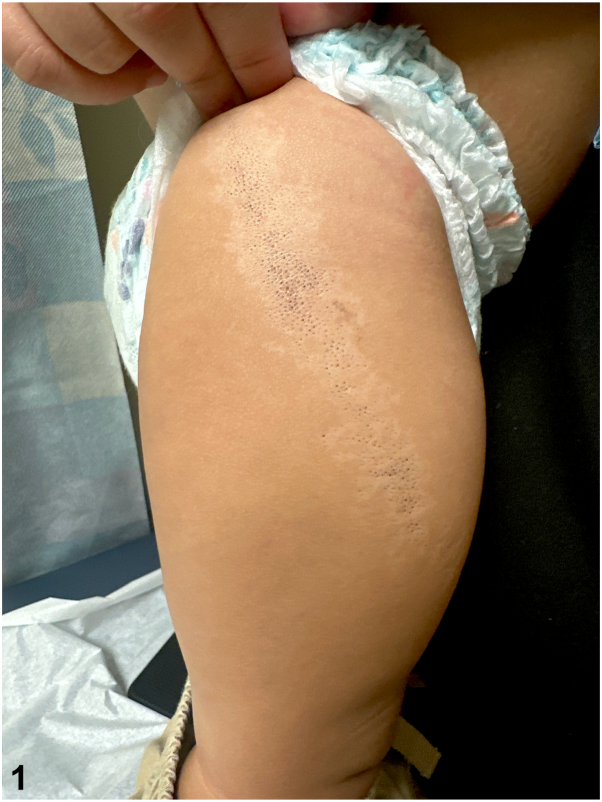

A 15-month-old male presented for evaluation of an isolated, asymptomatic lesion on the lateral aspect of his right thigh (Fig 1). The lesion was present since birth and increased in size proportional with the patient’s growth. He was otherwise healthy and not taking any medications.

**Question 1: What is the diagnosis?**

A. Inflammatory linear verrucous epidermal nevus (ILVEN)
B. Nevus comedonicus
C. Linear porokeratosis
D. Pigmentary mosaicism
E. Lichen striatus

**Answers:**

A. ILVEN – Incorrect. ILVEN is a clinical descriptor encompassing a heterogenous group of inflammatory skin disorders, such as linear psoriasis and porokeratosis. ILVEN may present at birth and classically follows a blaschkoid distribution. However, lesions consist of erythematous, verrucous, and scaly papules without follicular plugging. Furthermore, ILVEN is pruritic, which is inconsistent with our patient’s lack of symptoms.^1^
B. Nevus comedonicus – Correct. Our patient’s hypopigmented, blaschkoid patch containing comedonal-like openings is characteristic of nevus comedonicus. Nevus comedonicus is a subtype of epidermal nevus that may appear at birth or by 10 years of age. Clinical findings are characterized by grouped, dilated follicular ostia with keratin plugs resembling open comedones, or “blackheads.” Lesions may be in a linear or blaschkoid distribution, as seen in our patient.^2^
C. Linear porokeratosis – Incorrect. Although congenital linear porokeratosis is arranged along lines of Blaschko, patients present with erythematous papules and plaques with a keratotic rim; open comedones are not characteristic.^3^
D. Pigmentary mosaicism – Incorrect. Pigmentary mosaicism is genetic heterogeneity resulting in sharply demarcated, hypopigmented macules and patches following the lines of Blashko. Although our patient’s lesion fits this description, there are additionally numerous associated comedonal openings that are diagnostic of nevus comedonicus.^4^
E. Lichen striatus – Incorrect. Lichen striatus is yet another blaschkoid dermatitis. However, cutaneous findings consist of pink or hypopigmented papules that coalesce to form linear, scaly bands. These inflammatory changes would not be present at birth and were absent in our patient.^3^

**Question 2: What is the causative mutation?**

A. CARD14
B. IKBKG
C. PIK3CA
D. HRAS
E. FGFR2

**Answers:**

A. CARD14 – Incorrect. Approximately 10% of patients with ILVEN have an associated CARD14 mutation within blood and affected tissue. CARD14 mutations have not been linked to nevus comedonicus.^1^
B. IKBKG – Incorrect. Autosomal dominant mutations in the IKBKG gene (also known as NEMO), are responsible for the cutaneous lesions of incontinentia pigmenti. NEMO protein products confer protection against tumor necrosis factor-mediated apoptosis. Lesions present in various inflammatory and postinflammatory stages (vesicular, verrucous, hyperpigmentation, and hypopigmentation/atrophy) within a blaschkoid distribution.^4^
C. PIK3CA – Incorrect. PIK3CA gene’s protein product activates the mammalian target of rapamycin cell signaling pathway. Mutations in PIK3CA have been implicated in many inherited disorders, such as fibroadipose hyperplasia syndrome, which is associated with epidermal nevi and segmental overgrowth of skeletal and fibroadipose tissues, and CLOVES syndrome, which presents with a constellation of features including congenital lipomatous overgrowth, epidermal nevi, and scoliosis. Nevus comedonicus is not a reported association.^5^
D. HRAS – Incorrect. The HRAS gene is a member of the Ras family of oncogenes regulating cell division and apoptosis. HRAS mutations are associated with multiple diagnoses, such as woolly hair nevus, which consists of a well-demarcated area of hair with distinct texture, and phakomatosis pigmentokeratotica, an epidermal nevus syndrome characterized by nevus sebaceous and speckled lentiginous nevus. Nevus comedonicus is not a known component of phakomatosis pigmentokeratotica.^5^
E. FGFR2 – Correct. Genetic mosaicism due to mutations in fibroblast growth factor receptor 2, or FGFR2, have been implicated in nevus comedonicus. Interestingly, mutations in this gene are also responsible for Apert syndrome, an entity which is partially characterized by nodulocystic acne.^2^^,^^5^

**Question 3: Which is the most appropriate next step in treatment for this patient?**

A. Observation
B. Surgical resection
C. Topical corticosteroids
D. Oral isotretinoin
E. Pulse-dye laser therapy

**Answers:**

A. Observation – Correct. Nevus comedonicus is in itself a benign finding. Treatment, which can include topical or oral retinoids, keratolytics, or surgical excision, is usually pursued only for aesthetic reasons. In this very young patient, conservative observation is the most appropriate next step.^2^
B. Surgical resection – Incorrect. For this 15-month-old, surgical resection of this benign entity is unnecessary and carries risk of potential complications. Observation is more prudent.^2^
C. Topical corticosteroids – Incorrect. Our patient’s findings are asymptomatic and do not require pharmaceutical intervention. Topical corticosteroids could be considered if erythema or pruritus develops.^2^
D. Oral isotretinoin – Incorrect. Oral isotretinoin may be considered later if comedonal lesions become distressing or bothersome to the patient as he ages. Also, oral retinoids have been found to be largely ineffective in treating nevus comedonicus except in widespread inflammatory variants.^2^
E. Pulse-dye laser therapy – Incorrect. Pulse-dye laser is not an effective or first-line option for this patient. In single case reports, 2940-nm erbium-dosed yttrium aluminum garnet, 10,600-nm ultrapulse CO_2_, and 1450-nm diode lasers have shown improvement for nevi comedonicus, but with residual epidermal atrophy.^2^

## Conflicts of interest

None disclosed.
